# Mechanistic clues to the protective effect of chrysin against doxorubicin-induced cardiomyopathy: Plausible roles of p53, MAPK and AKT pathways

**DOI:** 10.1038/s41598-017-05005-9

**Published:** 2017-07-06

**Authors:** Eman M. Mantawy, Ahmed Esmat, Wesam M. El-Bakly, Rania A. Salah ElDin, Ebtehal El-Demerdash

**Affiliations:** 1 Department of Pharmacology & Toxicology, Faculty of Pharmacy, Ain Shams University, Cairo, Egypt; 2 Department of Pharmacology & Therapeutics, Faculty of Medicine, Ain Shams University, Cairo, Egypt; 3 Department of Anatomy, Faculty of Medicine, Ain Shams University, Cairo, Egypt

## Abstract

Doxorubicin (DOX) is the mainstay chemotherapeutic agent against a variety of human neoplasmas. However, its clinical utility is limited by its marked cardiotoxicity. Chrysin, is a natural flavone which possesses antioxidant, anti-inflammatory and anti-cancer properties. The current study aimed to investigate the potential protective effect of chrysin against DOX-induced chronic cardiotoxicity and the underlying molecular mechanisms. Male Sprague-Dawley rats were treated with either DOX (5 mg/kg, once a week) and/or chrysin (50 mg/kg, four times a week) for four weeks. Chrysin prevented DOX-induced cardiomyopathy which was evident by conduction abnormalities, elevated serum CKMB and LDH and histopathological changes. Chrysin also ameliorated DOX-induced oxidative stress by decreasing lipid peroxidation and upregulating the antioxidant enzymes. Moreover, chrysin attenuated DOX-induced apoptosis via decreasing expression of p53, Bax, Puma, Noxa, cytochrome c and caspase-3 while increasing expression of Bcl-2. DOX induced activation of MAPK; p38 and JNK and increased expression of NF-κB. Meanwhile, DOX suppressed AKT pathway via decreasing expression of its upstream activator VEGF and increasing expression of PTEN. Conversely, chrysin effectively neutralised all these effects. Collectively, these findings indicate that chrysin effectively protected against DOX-induced cardiomyopathy via suppressing oxidative stress, p53-dependent apoptotic pathway, MAPK and NF-κB pathways while augmenting the VEGF/AKT pathway.

## Introduction

Doxorubicin (DOX), an anthracycline antibiotic, is one of the most effective chemotherapeutic agents developed to date for treatment of a variety of cancers including solid tumors and hematologic malignancies^1^. Despite its therapeutic benefits, some restrictions have been imposed on its clinical efficacy because of the profound cardiotoxicity^2^. DOX cardiotoxicity is a complex multifactorial process which has been mainly associated with increased oxidative stress and mitochondrial abnormalities with subsequent cardiomyocyte apoptosis as a terminal downstream event^3^.

It is well known that intrinsic apoptotic pathway is initiated by the activation of p53 tumor suppressor gene which has been associated with DOX-induced cardiomyocyte apoptosis^4^. p53 can directly modulate the expression of a host of Bcl-2 family proteins which serve as crucial regulators of mitochondrial-dependent apoptotic pathway^5^. Bcl-2 proteins are divided into two main groups of proteins: a pro-apoptotic ones such as Bax and anti-apoptotic ones such as Bcl-2^6^. The activation of p53 switches the balance of the pro-apoptotic and anti-apoptotic members of the Bcl-2 family in favour of the pro-apoptotic ones^7^. These events trigger mitochondrial permeabilization, subsequent release of cytochrome c and activation of caspase enzymes which finally provoke apoptotic cell death^8^.

In this context, several signaling pathways have been implicated in regulating the intrinsic apoptotic pathway. Among them, nuclear factor kappa B (NF-kB) and mitogen-activated protein kinase (MAPK) are considered substantial intermediates for induction of apoptosis^9^. In contrast, several protein kinases provide important cell survival signals in the heart. Among them, the phosphoinositide 3-kinase (PI3-kinase)/Akt signaling pathway is probably the best characterized and most prominent pathway with regard to transmission of anti-apoptotic signals in cardiomyocytes survival^10^.

Based on this concept, several clinical and experimental studies have been directed toward employing various antioxidant and anti-apoptotic agents to prevent these detrimental cardiotoxic effects of DOX. Nowadays, much of attention has been paid to usage of phytochemicals as a promising approach to combat DOX-induced cardiotoxicity^11^.

Chrysin (5, 7-dihydroxyflavone) is present in several plant extracts, mushrooms (e.g. oyster mushroom), bee propolis and honey^12^. Chrysin is already available in market as a dietary supplement in the form of capsule (500 mg per capsule) and it is widely used as a sports supplement, with athletes taking doses up to 2–3 g per day without any associated side effects^13^. It has been shown to possess multiple biological properties such as antioxidant, anti-inflammatory, anti-apoptotic and anti-cancer^14^. chrysin has been proven to protect against different DOX toxicities including hepatotoxicity, nephrotoxicity^15^. Besides, in our lab we recently proved the cabability of chrysin pretreatment to protect against acute DOX cardiotoxicity in rats mainly due to its antioxidant, anti-inflammatory and anti-apoptotic effects^16^ Therefore, the current work is considered as an extension study where we are concerned with continuing studying the cardioprotective potential of chrysin but this time investigating chrysin co-treatment against the chronic form of DOX cardiotoxicity and elucidating the possible underlying molecular mechanisms with deeper focus on various signaling pathways implicated in cardiomyocytes death or survival.

## Results

### ECG

**Figure 1:**
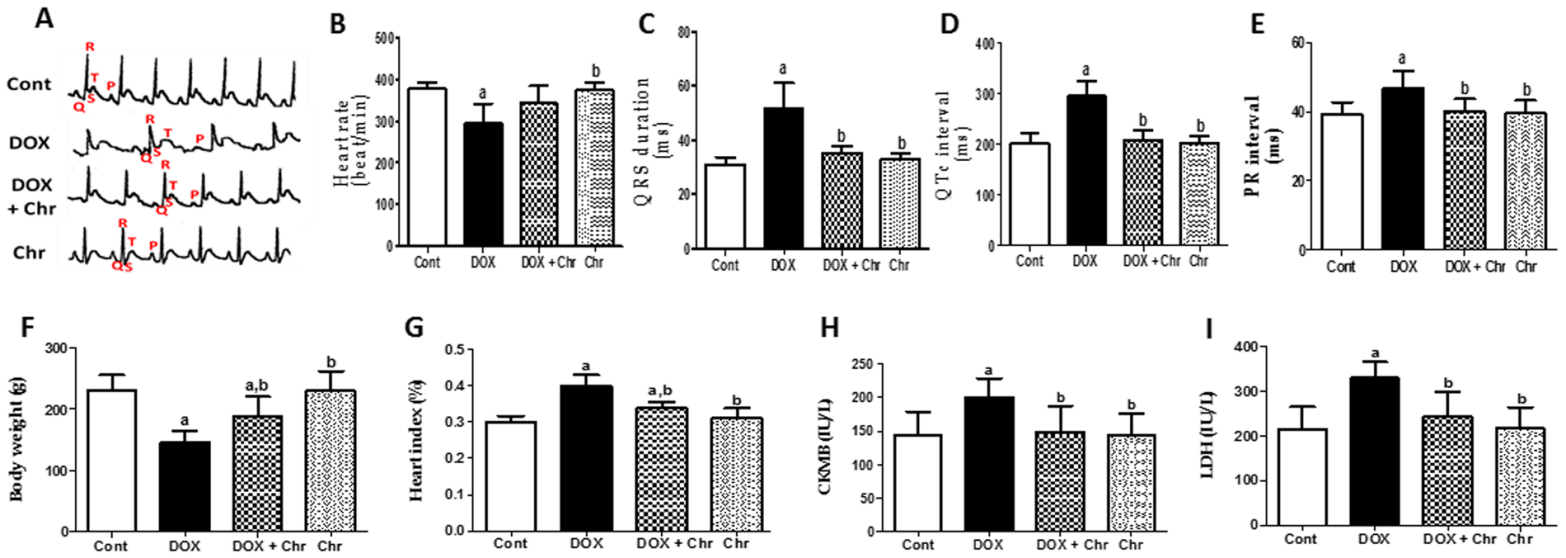
Effect of chrysin on ECG pattern, body and heart weight, and cardiotoxicity markers in rats subjected to chronic doxorubicin (DOX) intoxication. (**A**) ECG graph. (**B**) Heart rate (beat/min), (**C**) QRS duration (ms), (**D**) QTc interval (ms) (**E**) PR interval (ms). (**F**) Body weight (g), (**G**) Heart index (%), (**H**) CKMB activity (IU/L). And (**I**) LDH activity (IU/L). Data are represented as mean ± SD (n = 10). a or b: Statistically significant from the control or DOX group respectively at P < 0.05 using one-way analysis of variance ANOVA followed by Tukey-Kramer as a post-hoc test.

ECG tracing showed normal conduction properties in the control and chrysin only treated rats. Rats in DOX-treated group showed several ECG changes including bradycardia and prolongation of both QTc and PR intervals compared to the control group. Chrysin co-treeatment prevented the prolongation of both QTc and PR intervals. However, the heart rate in the group co-treated with DOX and chrysin was still statistically insignificant from both control and DOX group **(**Fig. 1A **)**.

### Cardiotoxicity indices

DOX intoxication led to a significant decrease in body weight reaching 37% compared to the control group indicating the growth impeding effect of DOX. On the other hand, the heart index significantly increased in the DOX-intoxicated group by 33% compared to the control group. On the contrary, co-treatment of intoxicated animals with chrysin effectively reversed these effects. Furthermore, the activities of serum markers indicating myocardial injury; creatine kinase isoenzyme-MB (CK-MB) and lactate dehydrogenase (LDH) were significantly elevated in the DOX-intoxicated group by 39 and 52% respectively compared to the control group. Co-treatment of intoxicated animals with chrysin significantly reduced the activities of both enzymes compared to DOX group. Chrysin alone treatment did not show any significant alterations in all these parameters when compared to the control group **(**Fig. 1B **)**.

### Histopathological examination

**Figure 2:**
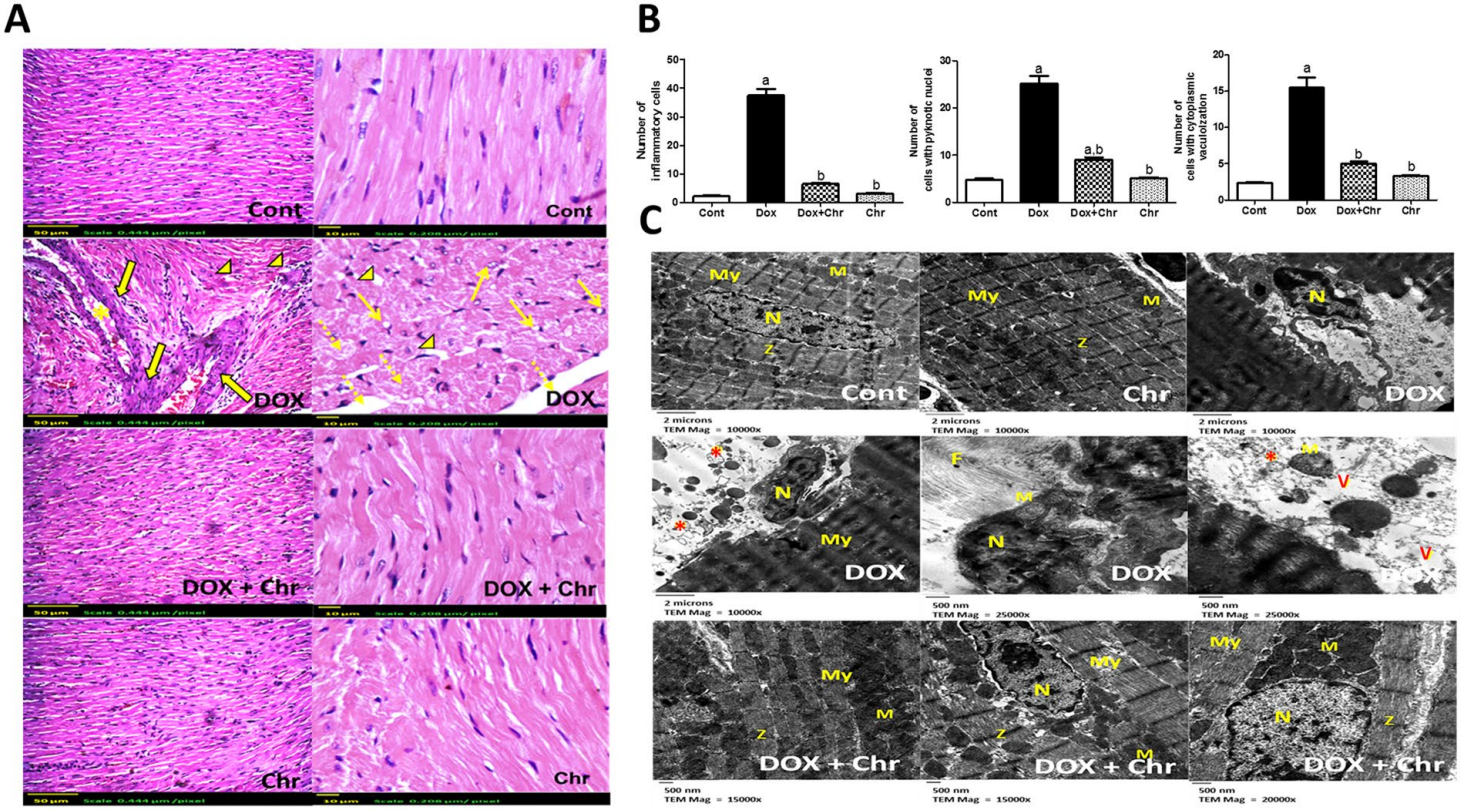
Effect of chrysin on histopathological alterations in rats subjected to chronic doxorubicin (DOX) intoxication. (**A**) Photomicrographs of heart sections stained by H & E, (arrows) pyknotic nuclei, (dotted arrows): myofibrillar loss, (arrowheads): cytoplasmic vacuolization, (solid arrows): inflammatory cell infiltration and (star): congestion. Scale bar, 50 µm (200x) and 10 µm (400x). (**B**) The mean number of inflammatory cells, cells with pyknotic nuclei and cells with cytoplasmic vacuolization in different treated groups. The measurements were done in 4 non overlapping fields in each of 5 different sections taken from 5 different animals of each group. Data are represented as mean ± SD (n = 10). a or b: Statistically significant from the control or DOX group respectively at P < 0.05 using one-way analysis of variance ANOVA followed by Tukey-Kramer as a post-hoc test. (**C**) Electronmicrographs of ultrathin sections of heart observed by transmission electron microscopy, (My): myofibril (M): mitochondria, (N): nucleus, (F): fibrosis, (asterisk): damaged mitochondria and (v): vacuoles. Scale bar, 2 µm (10000x) & 500 nm (15000, 20000 & 25000x).

Hearts from control and chrysin only treated rats showed regular cell distribution and normal myocardium architecture. Histological examination of hearts from DOX-intoxicated animals revealed marked myocardial degeneration in the form of myofibrillar loss, cytoplasmic vacuolization, inflammatory cell infiltration, edema, congestion and nuclear pyknosis. Interestingly, co-treatment of intoxicated rats with chrysin preserved the normal myocardium architecture (Fig. 2A **)**. The quantitative analysis for this histopathological findings revealed that DOX intoxication significantly elevated the no of each of inflammatory cells, cells with pyknotic nuclei and cells with cytoplasmic vacuolization by 12, 5 and 6 fold respectively compared to control group. While chrysin co-treatment significantly reduced the mean number of each cell type compared to DOX group **(**Fig. 2B).

Electron microscopic examination of cardiomyocytes of control and chrysin only treated groups revealed normal cardiac ultrastructure which is characterized by highly organized myofibrils with a normal striation pattern, abundant mitochondria with numerous cristas and a normally appearing rod-shaped nucleus. On the other hand, DOX intoxication caused dramatic ultrastructural changes in the cardiac cells in the form of myofibrillar disarray, loss of normal striation pattern, vacuolization, irregular folding of the nuclear surface and nuclear chromatin condensation and margination which are typical features of apoptosis. Besides, the mitochondria were condensed with inner wrinkled crista and showed heterogeneity both in size and shape. Additionally, some extracellular matrix is observed between the cardiomyocytes. Co-treatment of intoxicated animals with chrysin caused preservation of the ultrastructure of the cardiomyocytes in myofibrils, nuclei and mitochondrial morphology in comparison with DOX group **(**Fig. 2C).

### Oxidative stress markers and antioxidant enzymes

**Figure 3:**
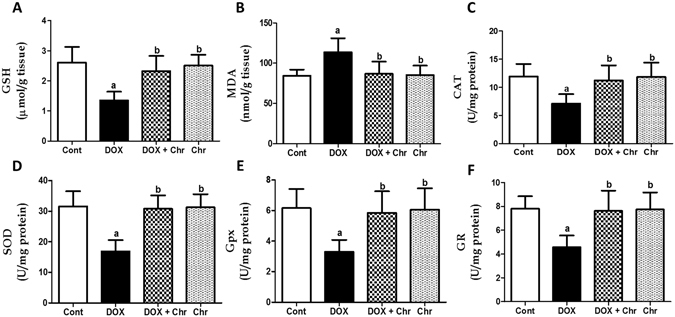
Effect of chrysin on oxidative stress markers and antioxidant enzymes in rats subjected to chronic doxorubicin (DOX) intoxication. (**A**) GSH (μmol/g tissue). (**B**) MDA (nmol/g tissue). (**C**) CAT (U/mg protein). (**D**) SOD (U/mg protein). (**E**) Gpx (U/mg protein). (**F**) GR (U/mg protein). Data are represented as mean ± SD (n = 10). a or b: Statistically significant from the control or DOX group respectively at P < 0.05 using one-way analysis of variance ANOVA followed by Tukey-Kramer as a post-hoc test.

DOX-induced redox imbalance in the heart was evaluated by assessing reduced glutathione (GSH) and malondialdehyde (MDA) levels in addition to various antioxidant enzyme activities in the cardiac tissues. DOX intoxicaion significantly reduced GSH level by 48% while increased lipid peroxides level by 34% compared to the control group. Moreover, DOX induced a significant decrease in the cardiac antioxidant enzyme activities; catalase (CAT), superoxide dismutase (SOD), glutathione peroxidase (Gpx) and glutathione reductase (GR) by 40, 47, 46 and 41% respectively, compared to the control levels. Meanwhile, co-treatment of intoxicated animals with chrysin afforded significant protection against DOX-induced oxidative stress as it restored the normal levels of both GSH and MDA and activities of the antioxidant enzymes. Additionally, animals treated with chrysin alone did not show any significant changes in both GSH and MDA levels and antioxidant enzyme activities compared to the control group **(**Fig. 3 **)**.

### Apoptotic markers

**Figure 4:**
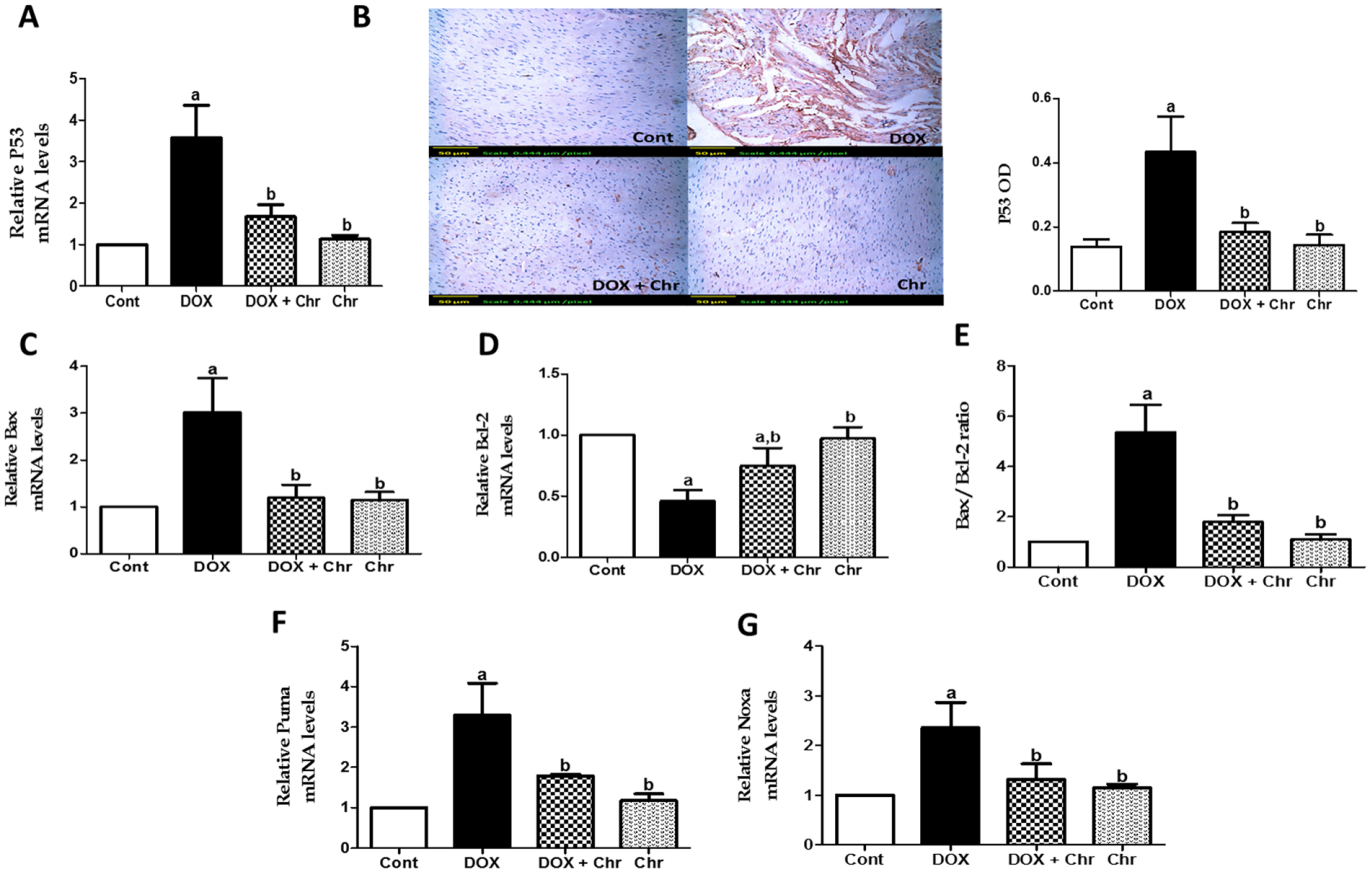
Effect of chrysin on expression of p53 and Bcl-2 family members in rats subjected to chronic doxorubicin (DOX) intoxication. (**A**) Gene expression of p53. (**B**) Protein expression of p53 by immunohistochemical staining. Scale bar, 50 µm (200x) and quantitative image analysis for immunohistochemical staining was expressed as optical densities (OD) across 10 different fields for each rat section. (**C**) Gene expression of Bax. (**D**) Gene expression of Bcl-2. (**E**) The ratio of Bax to Bcl-2 gene expression. (**F**) Gene expression of Puma G: Gene expression of Noxa. Quantitation of gene expression was done using real-time PCR. The levels of mRNA were normalized to that of GAPDH in each group. The mRNA levels of genes were expressed as relative quantification (RQ) compared to the control group. The value of genes in control group was defined as 1. Data are represented as mean ± SD (n = 10). a or b: Statistically significant from the control or DOX group respectively at P < 0.05 using one-way analysis of variance ANOVA followed by Tukey-Kramer as a post-hoc test.

p53 was assesed on both gene level using RT-PCR and protein level using immunohistochemistry. DOX treatment significantly increased p53 mRNA levels 3.5 fold compared to the control group. On the other hand, co-treatment of intoxicated animals with chrysin significantly reduced p53 gene expression compared to the DOX group **(**Fig. 4A **)**. Besides, DOX induced a marked elevation in the protein expression of p53 which was evident from the intense brown color compared to control group. While co-treatment of intoxicated animals with chrysin markedly diminished this elevated expression as manifested by the faint brown staining. The immunohistochemical staining was quantified as OD of the stained regions using the image analysis software **(**Fig. 4B **)**.

The gene expressions of Bcl-2 family members; (Bax, Bcl-2, Puma, Noxa) were assessed using RT-PCR. DOX intoxication significantly increased Bax mRNA levels by 3 fold while decreasing Bcl-2 mRNA levels by 0.54 fold compared to the control group. On the contrary, co-treatment of intoxicated animals with chrysin significantly decreased Bax expression while increased Bcl-2 expression compared to the DOX group. By statistically calculating the Bax to Bcl-2 ratio with respect to mRNA levels, DOX significantly increased the Bax/Bcl-2 ratio 5.36 fold compared to the control group while chrysin significantly decreased this elevated ratio compared to DOX group **(**Fig. 4C–E **)**. Additionally, DOX significantly increased mRNA levels of Puma and Noxa by 3.29 and 2.36 fold respectively compared to the control group. Meanwhile, co-treatment of intoxicated animals with chrysin significantly decreased the elevated expressions of both genes compared to the DOX group **(**Fig. 4F and G **)**.

**Figure 5:**
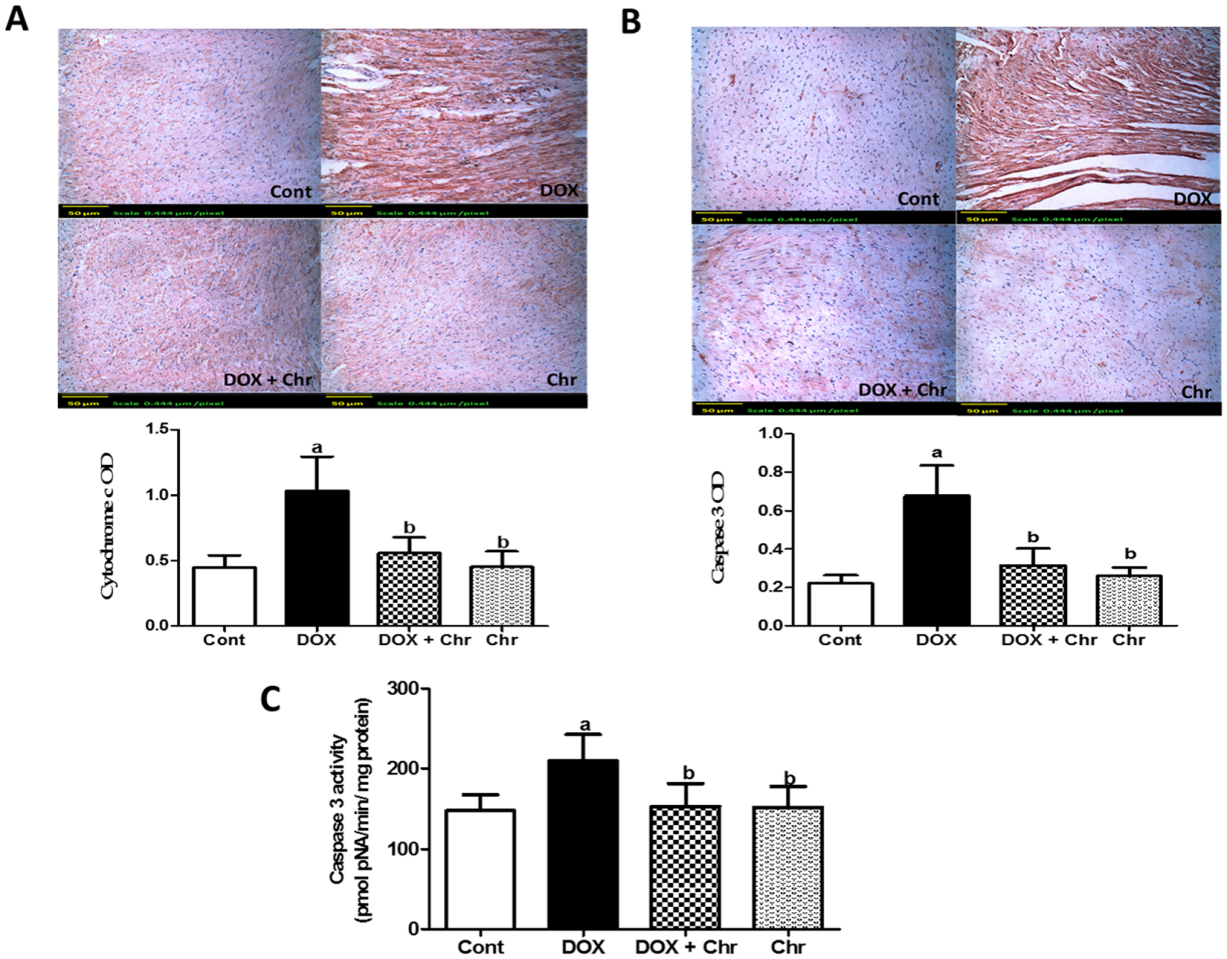
Effect of chrysin on cytochrome c and caspase 3 protein expressions and activity in rats subjected to chronic doxorubicin (DOX) intoxication. (**A**) Protein expression of cytochrome c by immunohistochemical staining. (**B**) Protein expression of caspase 3 by immunohistochemical staining. Scale bar, 50 µm (200x) and quantitative image analysis for immunohistochemical staining was expressed as optical densities (OD) across 10 different fields for each rat section. (**C**) Caspase 3 activity expressed as pmolpNA/min/mg protein. Data are represented as as mean ± SD (n = 10). a or b: Statistically significant from the control or DOX group respectively at P < 0.05 using one-way analysis of variance ANOVA followed by Tukey-Kramer as a post-hoc test.

Furthermore, the expression of cytochrome c protein was assessed using immunohistochemistry. DOX induced a marked increase in the expression of cytochrome c which was evident from the intensive brown color. Contrawise, co-treatment of intoxicated animals with chrysin markedly diminished this elevated expression. The immunohistochemical staining was quantified as OD of the stained regions using the image analysis software, **(**Fig. 5A **)**.

Induction of apoptotic machinery was finally confirmed by assessing both caspase-3 expression immunohistochemially and its activity colorimetrically. In the term of protein expression, DOX elevated the expression of the enzyme as shown by the intense brown staining compared to control group. While co-treatment of intoxicated animals with chrysin prevented this elevation to a large extent The immunohistochemical staining was quantified as optical density (OD) of the stained regions using the image analysis software. **(**Fig. 5B **)**.

Regarding the enzyme activity, DOX treatment significantly increased caspase 3 activity by 42% compared to the control group. While co-treatment of intoxicated animals with chrysin significantly decreased the enzyme activity compared to DOX group **(**Fig. 5C **)**.

### MAPK

**Figure 6:**
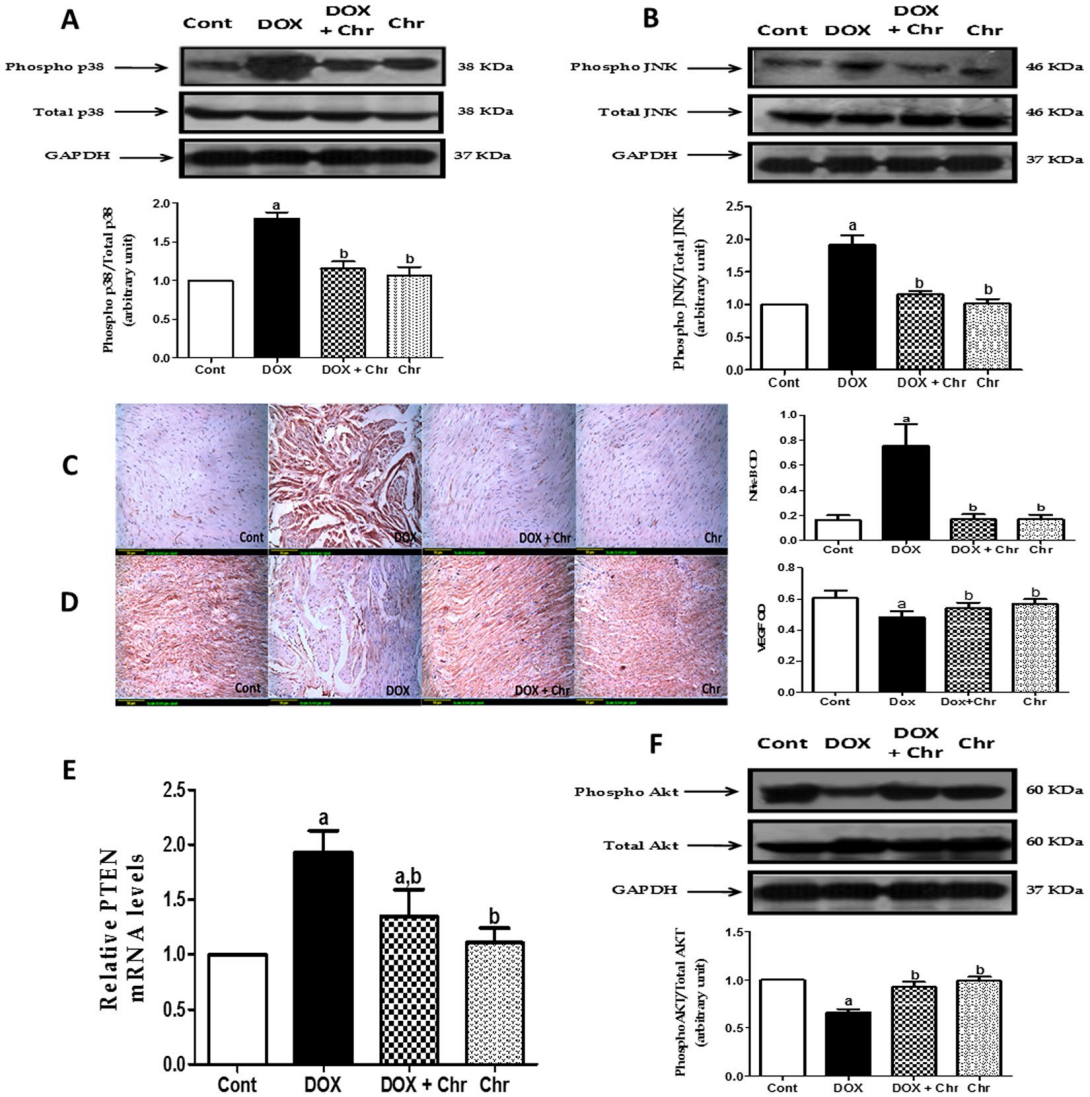
Effect of chrysin on NF-κB, MAPK, VEGF, PTEN and AKT expressions in rats subjected to chronic doxorubicin (DOX) intoxication. (**A**) Protein expression of phospho and total p38 by western blot analysis and densitometric quantitation of the bands (**B**) Protein expression of phospho and total JNK by western blot analysis and densitometric quantitation of the bands. Blot densitometry was performed using Image J analysis software. GAPDH was used as a loading control. (**C**) Protein expression of NF-κB by immunohistochemical staining (**D**) Protein expression of VEGF by immunohistochemical staining. Scale bar, 50 µm (200x) and quantitative image analysis for immunohistochemical staining was expressed as optical densities (OD) across 10 different fields for each rat section. (**E**) Gene expression of PTEN. Quantitation of gene expression was done using real-time PCR. The levels of mRNA were normalized to that of GAPDH in each group. The mRNA levels of genes were expressed as relative quantification (RQ) compared to the control group. The value of genes in control group was defined as 1. (**F**) Protein expression of phospho and total AKT by western blot analysis and densitometric quantitation of the bands. Blot densitometry was performed using Image J analysis software. GAPDH was used as a loading control. Data are represented as as mean ± SD (n = 10). a or b: Statistically significant from the control or DOX group respectively at P < 0.05 using one-way analysis of variance ANOVA followed by Tukey-Kramer as a post-hoc test.

To further elucidate the molecular mechanisms responsible for the protective effects of chrysin on DOX-induced cardiac cell death, the expression of both total and phosphorylated MAPK proteins; p38 and the c-Jun N-terminal kinases (JNK) were evaluated using an immunoblotting assay. As shown in Fig. 6A,B the protein level of phosphorylated p38 and JNK respectively were significantly increased by DOX without any change in total protein content of these kinases compared to the control group. On the contrary, co-treatment of intoxicated animals with chrysin significantly diminished the elevation of the phosphorylated protein levels of both p38 and JNK compared to the DOX group.

### Nuclear factor Kappa B (NF-κB)

NF-κB was assessed by detecting the activated subunit p65 in cardiac tissues using immunohistochemistry. DOX induced an increase in the p65 level in the cardiac tissues which was evident from intense brown staining. Conversely, co-treatment of intoxicated rats with chrysin significantly reduced the expression of NF-κB compared to DOX group. The immunohistochemical staining was quantified as optical density (OD) of the stained regions using the image analysis software **(**Fig. 6C).

### VEGF/AKT/PTEN pathway

To investigate the role of vascular endothelial growth factor (VEGF)/AKT/PTEN signaling pathway in the cardioprotective effect of chrysin, the protein expression of VEGF ligand was assessed using immunohistochemical tecchniques. Besides The gene expression of PTEN was studied using RT-PCR and the expression levels of both total and phosphorylated proteins of AKT were studied using an immunoblotting assay. DOX significantly increased the protein expression of VEGF in the cardiac tissues which was evident from intense brown staining. Meanwhile, co-treatment of intoxicated rats with chrysin significantly decreased this elevated expression compared to DOX group. The immunohistochemical staining was quantified as optical density (OD) of the stained regions using the image analysis software **(**Fig. 6D **)**. Additionally, DOX treatment significantly increased PTEN mRNA level 1.9 fold compared to the control group. On the other hand, co-treatment of intoxicated animals with chrysin could significantly reduced PTEN expression compared to the DOX group. **(**Fig. 6E **)**. Concerning AKT protein expression, DOX treatment significantly decreased the protein level of phosphorylated AKT without any change in total protein content compared to the control group. On the contrary, co-treatment of intoxicated animals with chrysin could significantly enhanced the phosphorylated levels of AKT comparedl to the DOX group **(**Fig. 6F **)**.

## Discussion

Despite the remarkable extensive research about DOX cardiotoxicity, the solution to this potentially fatal complication has not been explored yet. The present study aimed to investigate the potential protective effect of a pleiotropic flavonoid; chrysin against DOX-induced chronic cadriotoxicity in rats and to elucidate the underlyling molecular mechanisms of this potential cardioprotective effect.

DOX-induced cardiotoxicity was shown by ECG abnormalities in the form of bradycardia, QRS widening and prolongation of both QT and PR intervals. Moreover, DOX-induced myocardial damage was evidenced by the significant elevation of CK-MB and LDH enzymes activities. The aforementioned ECG abnormalities and biochemical findings were further confirmed by the severe myocardial degeneration as shown by histopathological examination of the cardiac tissues. On the contrary, chrysin co-treatment significantly blunted DOX-induced conduction and biochemical abnormalities and also preserved the normal architecture of cardiomyocytes to a great extent. This implies that chrysin effectively attenuated the progression of DOX- induced cardiotoxicity.

In the next part of the study, we investigated the potential molecular mechanisms underlying the cardioprotective effects of chrysin. The molecular pathogenesis of DOX-induced cardiomyopathy is still controversial. Indeed, oxidative stress is considered a major mechanism through which DOX injures the myocardium^17^. DOX induces free radicals generation mainly via the “redox cycling” of the anthracycline molecule^3^. One-electron reduction of the quinone moiety of DOX leads to formation of the corresponding semiquinone free radical. This rapidly reduces molecular oxygen producing reactive oxygen species (ROS) including superoxide and hydroxyl radicals and hydrogen peroxide^18^. These radicals cause marked oxidative damage of cellular and mitochondrial membranes as well as several cellular organelles obscuring their functions and leading ultimately to cell death^17^.

Additionally, one of the consequences of DOX-induced cardiac injury is exhaustion of the intracellular antioxidant enzymes thereby enhancing the cardiomyocytes vulnerability to oxidative damage^19^. In this context, heart is especially sensitive to oxidative damage due to plenty of mitochondria, the site of basal ROS generation and the inherent low antioxidant defenses that could be easily overwhelmed^20^. Indeed, many compound with potent anti-oxidant properties could effectively abbrogate DOX-induced cardiomyopathy^4, 21^.

In the current study, DOX-induced oxidative damage in cardiac tissues was evidenced by the markedly elevated lipid peroxidation products (MDA), depleted GSH levels and diminished activities of antioxidant enzyme; CAT, SOD, GPx and GR. On the other hand, chrysin supplementation effectively prevented DOX-induced redox imbalance in cardiac tissues. These findings revealed that the cardioprotective effect of chrysin against DOX cardiotoxicity may be attributed to attenuation of oxidative stress. Our results coincided with previous studies reporting the antioxidant potential of chrysin which could be attributed the free radical scavenging properties of the hydroxyl groups in the 5^th^ and 7^th^ position of chrysin^14, 22^.

Beside its direct deleterious effect, oxidative stress plays crucial roles in triggering mitochondrial-dependent apoptotic pathway which is the most direct cause of DOX cardiotoxicity^23^. ROS generated within the mitochondria during DOX metabolism leads to free radical attack of the mitochondrial membrane phospholipids resulting in loss of mitochondrial membrane potential and consequently release of cytochrome c into the cytosol^24^. Released cytochrome c forms complex with apoptosis protease activation factor-1 recruiting and activating the initiator caspase-9. This in turn activates the executer caspase-3 commencing the apoptotic degradation phase^25^.

Numerous studies have shown that DOX-induced apoptotic death of cardiomyocytes is associated with increased expression of p53 in response to both DNA damage and oxidative stress^4^. Activation of the intrinsic pathway of apoptosis depends on induction of p53 which further regulate the transcription of a host of target genes involved in apoptosis regulation notably, Bcl-2 family members which are the key controller of the mitochondrial membrane potential^5, 26^. On the basis of function, this family is divided into two main groups of proteins that elicit opposing effects on mitochondria, pro-apoptotic members and anti-apoptotic ones^6^.These proteins possess conserved BH domains where the anti-apoptotic members display sequence conservation through all BH domains while the pro-apoptotic members are further subdivided in two groups: multidomain and the BH3-only proteins^26^.

Among the multidomain pro-apoptotic members, Bax can augment apoptosis by perforating the outer mitochondrial membrane in response to oxidative stimuli increasing membrane permeability and subsequntely triggering cytochrome c release^8^. Besides, BH3-only proteins of the Bcl-2 family such as Puma and Noxa can also contribute to p53-mediated apoptosis via indirect induction of mitochondrial outer membrane permeabilization^27^. As in the cytosol, anti-apoptotic Bcl-2 protein sequester Bax protein to inhibit its pro-apoptotic function and translocation to the mitochondria^28^. Puma and Noxa relieves this inhibition of the pro-apoptotic Bax through their direct binding with the prosurvival Bcl-2 proteins thus inactivating them^27^. Upon activation, p53 transcriptionally upregulates the gene expression of the pro-apoptotic members including Bax, Puma and Noxa while downregulates the gene expression of the anti-apoptotic Bcl-2 thereby activates the intrinsic apoptotic pathway^29, 30^.

In this regard, several studies showed that many compounds attenuated DOX-induced cardiomyocytes cell death via suppressing p53 signaling pathway thus represent a prominent target for protection against DOX cardiotoxicity^4, 31^.

Our results indicated that DOX intoxication elicited a series of apoptotic events in the mitochondrial apoptotic pathway as shown by the significantly increased p53 and cytochrome c protein expression, upregulated pro-apoptotic members; Bax, Puma and Noxa, downreguled anti-apoptotic Bcl-2 gene and elevated caspase 3 expression and activity. In this context, our results are in accordance with previous studies that reported mitochondrial dysfunction due to DOX-induced oxidative stress^24^. In this regard, many studies proved the ability of DOX to downregulate p53 gene expression^32^. Moreover, several studies have reported an increase in Bax and a decrease in Bcl-2 gene expression in cardiomyocytes following DOX intoxication^11, 33^. In addition, it was shown that DOX increased puma expression which contributes to DOX apoptotic cell death in both cardiomyocytes and various cancer cell lines^34^. Despite, there is no reports about DOX effects on Noxa expression in heart, it was reported that DOX-induced Noxa expression plays critical role in DOX-induced apoptosis in neuroblastoma cells ^35^. Interestingly, DOX-induced alteration in expression of p53, cytochrome c, Bax, Puma, Noxa, Bcl-2 and caspase-3 expression and activity were restored to normal levels with chrysin supplementation. These findings proved the anti-apoptotic effect of chrysin which is consistent with previous studies reporting the ability of chrysin to guard against apoptosis mainly via suppressing p53 signaling pathway^36, 37^.

To further explore the molecular mechanisms underlying the anti-apoptotic effects of chrysin, MAPK, NF-kB and VEGF/PI3K/Akt signaling pathways were investigated. Several lines of evidence indicate that MAPK are key intermediates in induction of the mitochondrial-dependent apoptotic pathway^9^. MAPK are serine/threonine protein kinases that consists of three major signaling cascades: the extracellular signal-related kinases (ERK1/2), JNK, and the p38 kinase (p38)^38, 39^. Growing evidence reported that ROS activate both JNK and p38 MAPK which consequently contribute to induction of apoptosis^11^. These kinases can trigger apoptosis through specific phosphorylation and hence activation of downstream mediators of apoptosis such as p53 and Bax^31, 40^. Besides, it was reported that p38 induces phosphorylation of Bcl-2 thereby enhances its proteasomal degradation^41^.

In the present study, DOX increased the phosphorylation of both kinases; p38 and JNK. Our results coincided with previous studies showing enhanced phosphorylation and activation of p38 and JNK in various models of DOX cardiotoxicity^9, 11, 31^. Conversely, chrysin supplementation could effectively reversed this DOX-induced p38 and JNK activation. These findings agreed with a previous study reporting the inhibitory effect of chrysin on MAPK signaling pathway that contributed to protective effect of chrysin against cisplatin-induced apoptotic cell death in colon tissues^37^.

One of the major signal-transduction pathways that is activated in response to oxidative stress is NF-κB which plays crucial rules in cell survival, inflammation and immune responses^42^. Recent studies have shown that NF-κB augments DOX-induced apoptosis in vascular cells and myocytes^9, 43^. The pro-apoptotic properties of NF-κB could be attributed to upregulating the expression of some apoptotic genes including p53 and Puma while downregulating the expression of some anti-apoptotic ones, e.g. Bcl-XL^34, 43^. Recently, it was found that JNK and p38 MAPK play roles in DOX-induced NF-kB activation^9^. In the present study, NF-κB expression was markedly elevated in the DOX-intoxicated animals while chrysin supplementation effectively counteracted this effect. Our results are in accordance with pervious one reporting that downregulating NF-κB protein expression could be a promising target for protection against DOX cardiotoxicity^32^.

Accumulating evidence indicates the PI3K /Akt signaling pathway is a crucial cell survival pathway in cardiomyocytes^10^. This pathway is activated by a variety of growth factors such as VEGF^44^. VEGF has been reported to prevent the ongoing loss of muscle mass in the hypertrophied myocardium by blunting apoptotic cardiomyocyte death^45^. VEGF activates receptor tyrosine kinases leading to activation of various signal transducers including PI3-K^46^. PI3K is a heterodimeric enzyme that catalyzes the production of the lipid secondary messenger phosphatidylinositol-3,4,5-triphosphate, which in turn activates a wide range of target proteins including the serine/threonine kinase AKT^47^. Once activated, Akt proceeds to phosphorylate several downstream targets which mediate its anti-apoptotic effects such as Bad and caspase 9 thus inactivates them and suppresses the intrinsic pathway of apoptosis^48^. Negative regulation of the PI3K/AKT pathway is primarily accomplished through the action of the phosphatase enzyme; PTEN tumor suppressor protein which functions by dephosphorylating phosphatidylinositol-3,4,5-triphosphate thereby inhibiting this survival pathway^49^.

In this regard, it was reported that there is a mutual crosstalk between the AKT and p53 pathways^50^. AKT has been shown to negatively regulate p53 transcriptional activity through its substrate MDM2 which is an E3 ubiquitin ligase essential for ubiquitination and subsequent proteosomal degradation of p53^51^. Phosphorylation of MDM2 by AKT stimulates its translocation to the nucleus, where it binds to p53 promoting its degradation^48^. Meanwhile, it has been reported that p53 induces PTEN gene expression thus repressing AKT activation^52^.

The current study showed that DOX increased the PTEN gene expression while decreased VEGF expression and the phosphorylation of AKT protein. On the contrary, chrysin could effectively prevent these effects. In this context, several studies have shown that DOX cardiotoxicity is associated with reduced phosphorylation of AKT protein and hence downregulation of this survival pathway^9, 53^. In addition, upregulating VEGF expression through transfecting cardiomyocytes with adenovirus vector encoding the gene mitigates DOX-induced apoptotic cell death via up regulating AKT signaling^54^ pathway. Despite lack of studies regarding the effects of DOX on PTEN in the heart, it has been recently reported that DOX increases PTEN gene expression which contributes to the apoptotic effects of DOX in the gastric cancer cells^55^.

**Figure 7:**
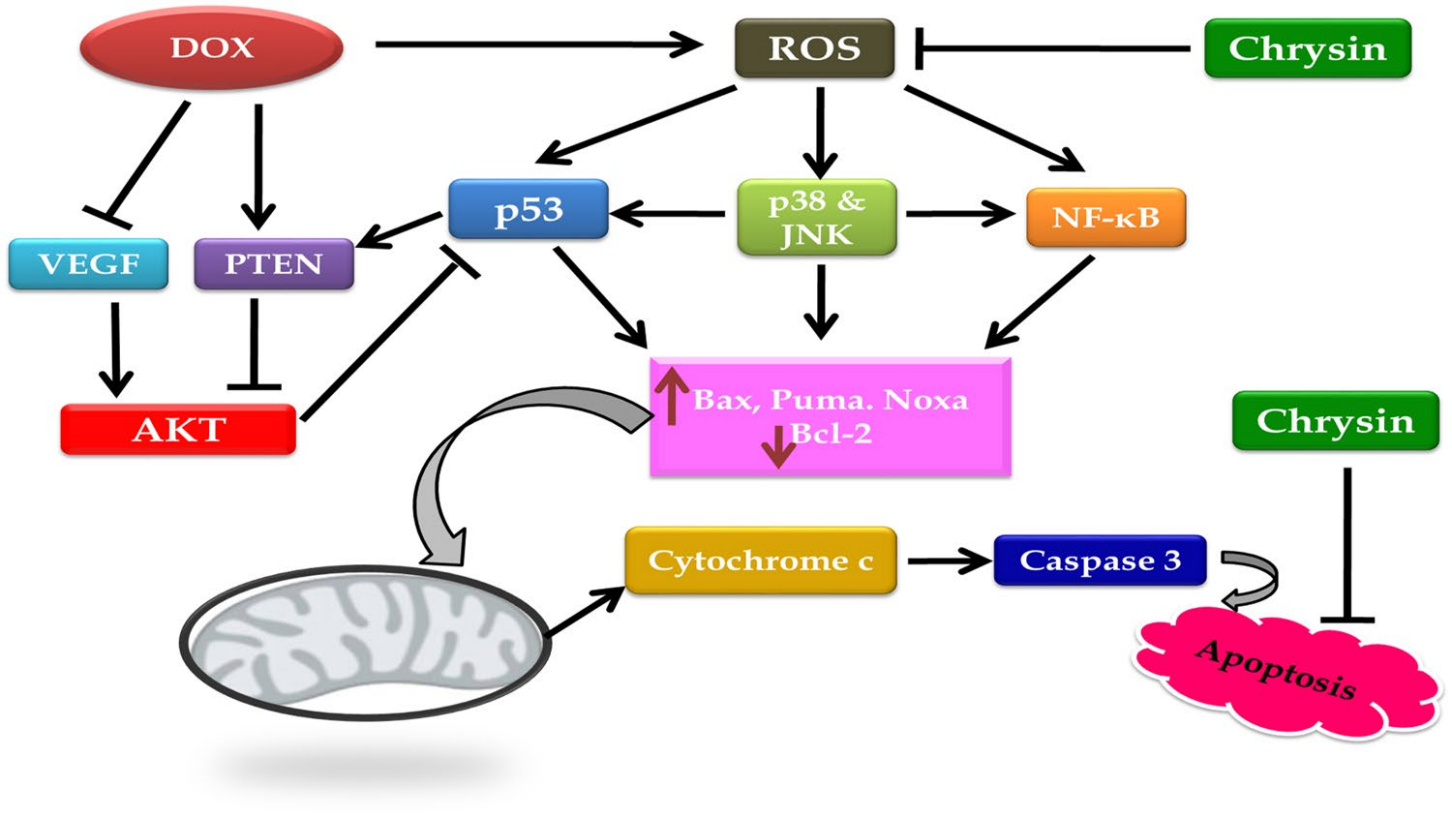
Schematic diagram of the signal transduction pathways implicated in the cardioprotection of chrysin against DOX-induced cardiomyopathy.

In conclusion, our findings outline some mechanistic insights into how chrysin protects against DOX-induced cardiomyopathy **(**Fig. 7 **)**. Firstly, it guards against DOX-induced oxidative stress via restoring cellular defense mechanisms. Secondly, it inhibited DOX-induced mitochondrial-dependent-apoptotic cell death by suppressing p53, p38, JNK and NF-κB signaling pathways. Finally, it augmented the VEGF/PI3K/Akt signaling pathway thus promoted the cardiomyocytes survival. These findings indicate that chrysin may be a novel adjunct therapy with DOX for mitigation of its potentially fetal cardiac complications.

## Materials and Methods

### Drugs and chemicals

DOX was purchased as Adriablastine (50 mg doxorubicin hydrochloride, Pharmacia & Upjohn, Milan, Italy). Chrysin, GSH, Ellman’s reagent [5,5-dithio-bis (2-nitrobenzoic acid); DTNB], bovine serum albumin, dimethylsulphoxide (DMSO) and thiobarbituric acid were purchased from Sigma Chemical Co. (St Louis, MO, USA). Anti total p38, anti phospho p38, anti total JNK and anti phospho JNK antibodies were purchased from R & D systems (Minneapolis, USA). Anti total AKT and anti phospho AKT antibodies were purchased from Biovision Co. (San Francisco, USA). Anti GAPDH antibody was purchased from Santa cruz biotechnology (Santa cruz, Texas, USA). Anti NF-κB p65, VEGF, p53, cytochrome c, caspase 3 antibodies were purchased from Thermo Fisher Scientific Co. (Waltham, MA, USA). All other chemicals were of the highest purity grade commercially available.

### Animals

Male Sprague-Dawley rats (150–250 g) were obtained from Nile Co. for Pharmaceutical and Chemical industries, Egypt. Rats were housed in an air-conditioned atmosphere at a temperature of 25 °C with alternatively 12 h light and dark cycles and allowed free access to food and water. Animals were acclimated for 2 weeks before experimentation. They were kept on a standard diet and water *ad libitum*. Standard diet pellets (El-Nasr, Abu Zaabal, Egypt) contained not less than 20% protein, 5% fiber, 3.5% fat, 6.5% ash and a vitamin mixture.

### Ethics Statement

Animal care and all experimental protocols were approved and conducted in accordance with the guidelines approved by the Research Ethics Committee of Ain Shams University (REC-ASU), Egypt.

### Experimental design

Rats were randomly assigned to four groups (ten animals per group) and treated for four weeks as following; The first group (control group) received 2.5 ml/kg of mixture of DMSO and corn oil (1:9) (vehicle for chrysin) through oral gavage four times weekly and i.p injection of saline (vehicle for DOX) once weekly. The second group received an oral dose of mixture of DMSO and corn oil (1:9) four times weekly and i.p injection of DOX (5 mg/kg) once weekly. The third group received chrysin 50 mg/kg through oral gavage four times weekly and i.p injection of DOX (5 mg/kg) once weekly. The last group received chrysin only at a dose of 50 mg/kg four times weekly.

Forty-eight hours after last chrysin administration, rats were anesthetized with ketamine (75 mg/kg; i.p.) and subjected to ECG recording. Blood samples were collected from the retro-orbital plexus and allowed to clot. Serum was separated for biochemical analyses. The body and heart weights were measured. Then, rats were sacrificed and heart tissues were dissected and washed with ice-cold saline. The whole cardiac tissues were used for assessing studied parameters to evaluate the cardiotoxic effects of doxorubicin on the whole heart tissues as done in earlier studies^9, 56^. Hearts were homogenized in saline then the homogenate was used for assessment of different biochemical parameters. In addition, heart specimens from different groups were used for histopathological and immunohistochemical examination.

### Electrocardiography (ECG)

ECG was recorded in ketamine anesthetized rats using Bioscience ECG recorder (Bioscience, Washington, USA). Needle electrodes were inserted beneath the skin for the limb lead at position II. Analysis of ECG waves was done to calculate heart rate (beats/min), QRS duration (ms), QT interval (ms), which was corrected for heart rate using the formula [QTc = QT/(square root of RR interval)] and PR interval (ms).

### Assessment of cardiotoxicity indices

CK-MB and LDH activities were determined according to standard methods using available commercial kits (Spectrum diagnostics, Cairo, Egypt). Heart index was calculated according to the formula: (heart weight/body weight) × 100.

### Histopathological examination

For light microscopy, heart specimens were fixed in 10% formalin and processed for paraffin sections of 4 μm thickness. Sections were stained with Hematoxylin and Eosin (H&E) and examined under a light microscope (Olympus BX-50). The mean numbers of inflammatory cells, cells with pyknotic nuclei and cells with cytoplasmic vacuolization were counted in the different groups using the image analyser Leica Q500 MC program.

For electron microscopic examination, heart specimens were immediately fixed in 2.5% phosphate buffered gluteraldehyde (pH 7.4) at 4 °C for 24 h and post fixed in 1% osmium tetraoxide for 1 h then dehydrated in ascending grades of ethanol. After immersion in propylene oxide, the specimens were embedded in epoxy resin mixture. Semithin sections (1 μm thickness) were cut, stained with toluidine blue and examined by light microscopy. Ultrathin sections (80–90 nm thickness) were stained with uranyl acetate and lead citrate then examined and photographed with JEOL 1010 transmission electron microscopy.

### Assessment of oxidative stress markers and antioxidant enzyme activities

Assessment of levels of GSH and MDA and activities of CAT, SOD, GPx and GR in heart homogenate was done using kits provided by Biodiagnostics, Giza, Egypt following the protocol provided by the manufacturer. The enzyme activities were expressed as U/mg protein. Protein was determined according to method of Lowry *et al*.^57^.

### Assessment of caspase 3 activity

Caspase-3 activity was assayed using a kit purchased from Sigma Chemical Co. (St Louis, MO, USA) following the protocol provided by the manufacturer.

### Real-time PCR

The mRNA levels of p53, Bax, Bcl-2, Puma, Noxa, and PTEN were measured by the real-time RT-PCR method using SYBR green. Total RNA was extracted from the cardiac tissues using TRIzol reagent and RNeasy Mini kit (Qiagen, CA, USA) according to the manufacturer’s instructions. Approximately 1 µg of total RNA was reverse transcribed in a reaction volume of 20 µL using a High-Capacity cDNA Reverse Transcription Kit (Life Technology, Carlsbad, California, USA). The primer sequences used in the PCR amplification processes are shown in Table 1. RT-PCR was carried out using SYBR green PCR Master Mix kit (Life Technology, Carlsbad, California, USA) in an Applied Biosystems Step One real-time quantitative PCR instrument. Thermal cycling conditions included one cycle of 95 °C for 10 min for activating hot-start Taq DNA polymerase followed by 45 cycles of denaturation (95 °C, 15 s) and combined annealing/extension (60 °C, 1 min). Gene expression changes were calculated by the comparative Ct method. The mRNA levels were normalized to the housekeeping gene, glyceraldehyde-3-phosphatedehydrogenase (GAPDH).

**Table 1:** Sequence of primers used in RT-PCR.

Target genes	Primer pairs (5′-3′)	
P53	Forward	5′-CAGCTTTGAGGTTCGTGTTTGT-3′
	Reverse	5′-ATGCTCTTCTTTTTTGCGGAAA-3′
Bax	Forward	5′-GATCAGCTCGGGCACTTTA-3′
	Reverse	5′-TGTTTGCTGATGGCAA CTTC-3′
Bcl-2	Forward	5′-AGGAT TGTGG CCTTC TTTGA GT-3′
	Reverse	5′-GCCG GTTC AGG TACT CAGT CAT-3′
Puma	Forward	5′- GTG TGG AGG AGGAGG AGT GG -3′
	Reverse	5′-TCG GTG TCG ATG TTG CTC TT-3′
Noxa	Forward	5′-GAACG CGCCA TTGAA CCCAA-3′
	Reverse	5′-CTTTG TCTCC AATTC TCCGG-3′
PTEN	Forward	5′-CAATGTTCAGTGGCGGAACTT-3′
	Reverse	5′-GGCAATGGCTGAGGGAACT-3′
GAPDH	Forward	5′-TCCCTCAAGATTGTCAGCAA-3′
	Reverse	5′-AGATCCACAACGGATACATT-3′

### Western blotting1

Heart tissue were homogenized in ice-cold RIPA lysis buffer (0.22% Beta glycerophosphate, 10% Tergitol-NP40, 0.18% Sodium orthovanadate, 5% Sodium deoxycholate, 0.38% EGTA, 1% SDS, 6.1% Tris, 0.29% EDTA, 8.8% Nacl, 1.12% Na pyrophosphate decahydrate) (Abcam, MA, USA) containing protease and phosphatase inhibitor cocktails (Roche Diagnostic, IN, USA). The protein concentration was determined using bicinchoninic acid protein assay kit (Biovision Co.,San Francisco, USA). Equal amounts of proteins were separated under denaturing conditions using 10% SDS-PAGE and electrophoretically transferred to a polyvinylidene difluoride membrane (Bio-Rad Laboratories Hercules, California, USA). The membranes were immunoblotted with one of the following primary antibodies; rabbit polyclonal anti-total JNK, rabbit polyclonal anti-phospho JNK, rabbit polyclonal anti-total p38, rabbit polyclonal anti-phospho p38, rabbit polyclonal anti-total AKT or rabbit polyclonal anti-phospho AKT. Each primary antibody was diluted in tris buffered saline tween (TBST) containing 0.5% fat-free milk powder. Then membranes were incubated with appropriate horseradish peroxidase–conjugated secondary antibody in TBST containing 0.5% fat-free milk powder. GAPDH served as the loading control. Bands were detected by enzyme-linked chemiluminescence reagent (Abcam, MA, USA) and developed to HyBlot CL autoradiography film. Blot densitometry was performed and bands were analyzed with Image analysis software (Image J, 1.46a, NIH, USA). The specific activity of phospho p38, phospho JNK and phospho-AKT were calculated as a ratio of total p38, JNK and AKT respectively.

### Immunohistochemistry

Paraffin embedded tissue sections of 3 μm thickness were rehydrated first in xylene and then in graded ethanol solutions. The slides were then blocked with 5% bovine serum albumin in TBS for 2 h. The sections were then immunostained with one of the following primary antibodies; rabbit polyclonal anti VEGF antibody rabbit polyclonal anti NF-κB p65 antibody, mouse monoclonal anti p53 antibody, mouse monoclonal anti-cytochrome c antibody or rabbit polyclonal anti caspase 3 antibody at a concentration of 1 μg/ml containing 5% bovine serum albumin in TBS and incubated overnight at 4 °C. Then sections were incubated with goat anti-rabbit secondary antibody. Afterthat, sections were incubated for 5–10 min in a solution of 0.02% diaminobenzidine containing 0.01% H_2_O_2_. Negative controls were performed in which the primary antibody was substituted with the normal rabbit IgG. Counter staining was performed using hematoxylin. Immunohistochemical quantification was carried out using image analysis software (Image J, 1.46a, NIH, USA).

### Statistical analysis

Data are presented as mean ± S.D. Multiple comparisons were performed using one-way ANOVA followed by Tukey-Kramer as a post-hoc test. The 0.05 level of probability was used as the criterion for significance. All statistical analyses were performed using Instat version 3 software package. Graphs were sketched using GraphPad Prism (ISI® software, USA) version 5 software.
